# Early and Mid-Term Results of Endovascular Aneurysm Repair with the Cordis Incraft Ultra-Low Profile Endograft: A High-Volume Center Experience

**DOI:** 10.3390/jcm13185413

**Published:** 2024-09-12

**Authors:** Luigi Baccani, Gianbattista Parlani, Giacomo Isernia, Massimo Lenti, Andrea Maria Terpin, Gioele Simonte

**Affiliations:** Vascular and Endovascular Surgery Unit, S. Maria della Misericordia University Hospital, 06132 Perugia, Italy; parlani.gianbattista@gmail.com (G.P.); iserniagiacomo1@gmail.com (G.I.); massimo.lenti@gmail.com (M.L.); andreamariaterpin@gmail.com (A.M.T.); giosimonte@gmail.com (G.S.)

**Keywords:** endograft, endovascular aortic repair, EVAR, low profile, hostile access

## Abstract

**Background/Objectives**: In recent years, manufacturers have developed new low-profile stent grafts to allow endovascular treatment of abdominal aortic aneurysms (AAA) in patients with small access vessels. We evaluated the early and mid-term outcomes of the Incraft (Cordis Corp, Bridgewater, NJ, USA) ultra-low profile endograft implantation in a high-volume single center. **Methods**: Between 2014 and 2023, 133 consecutive endovascular aneurysm repair (EVAR) procedures performed using the Incraft endograft were recorded in a prospective database. Indications included infrarenal aortic aneurysms, common iliac aneurysms, and infrarenal penetrating aortic ulcers. Mid-term results were analyzed using the Kaplan–Meier method. **Results**: During the study period, 133 patients were treated with the Cordis Incraft endograft, in both elective and urgent settings. The Incraft graft was the first choice for patients with hostile iliac accesses, a feature characterizing at least one side in 90.2% of the patients in the study cohort. The immediate technical success rate was 78.2%. The intraoperative endoleak rate was 51.9% (20.3% type 1 A, 0.8% type 1 B, and 30.8% type 2 endoleak). Within 30 days, technical and clinical success rates were both 99.3%; all type 1A and 1B endoleaks were resolved at the 30-day follow-up CT-angiogram. After a mean follow-up of 35.4 months, the actuarial freedom from the re-intervention rate was 96.0%, 91.1%, and 84.0% at 1, 3, and 5 years, respectively. The iliac leg patency rate was 97.1%, 94.1%, and 93.1% at 1, 3, and 5 years, respectively. No statistically significant differences were observed between hostile and non-hostile access groups, nor between the groups with grade 1, grade 2, and grade 3 access hostility. **Conclusions**: The ultra-low profile Cordis Incraft endograft represents a valid option for the endovascular treatment of AAA in patients with hostile iliac accesses. The procedure can be performed with high rates of technical and clinical success at 30 days and the rates of iliac branch occlusion observed during the follow-up period appear acceptable in patients with poor aorto-iliac outflow.

## 1. Introduction

Since the first report of successful endovascular aneurysm repair (EVAR) achieved in 1991 [1], EVAR has become the preferred treatment modality for abdominal aortic aneurysm (AAA) repair in most patients with suitable anatomy and reasonable life expectancy [2].

Anyway, for most patients with long life expectancy, open surgical repair should be considered a valid and absolutely appropriate option for elective AAA repair [2].

The main advantages of EVAR over open surgery repair (OSR) include lower mortality and morbidity rates in the peri-operative period, reduced operating time, the possibility of performing the operation under local anesthesia, and shorter post-operative in-hospital stays.

However, the endovascular approach requires the patient to be enrolled in longitudinal follow-up protocols for long-term surveillance, as some endograft-related complications may occur over time, such as endoleaks, migration, endograft rupture or disconnection, and iliac leg occlusion leading to acute limb ischemia [3,4,5].

Despite these potential disadvantages, the lower complication rates compared to OSR, especially in the short and mid-term period, and the continuous technological advancements that provide increasingly high-performance devices have led to a growing preference for this type of strategy, sometimes extending the indications to more challenging cases.

The aim of the present study was to investigate the early and mid-term results achieved during almost 9 years of experience in a high-volume center with the Cordis Incraft (Cordis Corp, Bridgewater, NJ, USA) ultra-low profile endograft for the treatment of abdominal aortic aneurysms.

## 2. Materials and Methods

Data from all 133 consecutive patients undergoing abdominal aortic aneurysm repair, both in elective and urgent settings, using the Cordis Incraft endograft from December 2014 to July 2023 at a single center were reviewed retrospectively.

Patient characteristics, pre-operative and intra-operative data, clinical events, and follow-up data were retrieved from a prospective electronic database filled at the time of the intervention and afterward during any scheduled examination or new hospitalization.

Indications for intervention included the presence of infrarenal aortic aneurysm with axial diameter ≥ 50 mm; common iliac aneurysm ≥ 35 mm; and infrarenal penetrating aortic ulcer.

In all cases, EVAR implantation was planned after evaluating the pre-operative computed tomography (CT) angiogram using a dedicated workstation (Aquarius Terarecon, Foster City, CA, USA).

The Cordis Incraft was always considered the first choice endograft for patients with hostile vascular access anatomy (e.g., small diameter of the iliac axis, tortuosity, circumferential calcifications, severe iliac stenosis or occlusions, and narrow aortic bifurcation).

All procedures were performed by a dedicated team of vascular surgeons under local or general anesthesia; the intervention took place in a hybrid operating room equipped with a fixed ceiling-mounted X-ray imaging system with a flat panel detector. A completion angiogram was always performed at the end of the procedure to evaluate endograft patency and aneurysm exclusion.

Intraoperative results were evaluated in terms of technical success, other associated procedures, the necessity of surgical conversion of the accesses, the presence of an endoleak on the final angiogram, limb ischemia, bleeding, and endograft occlusion.

Based on the most recent reporting standards [6], technical success was defined as correct advancement and deployment of the endograft without any of the following: intraoperative death; intra-procedural conversion into OSR; the presence of type I or type III endoleaks on the final angiogram; or intraoperative endograft or iliac leg occlusion.

The 30-day results were evaluated in terms of clinical success, mortality, need for reintervention, need for open surgical conversion, and endoleak.

Clinical success was considered achieved in all patients who, at 30 days post-EVAR procedure, met all the following criteria: technical success; absence of death from the initial procedure, secondary intervention, or aorta-related cause; absence of persistent type I or type III endoleak; absence of aneurysm sac expansion > 5 mm; absence of device migration > 10 mm; absence of failure due to device integrity issues; absence of aneurysm rupture; absence of conversion to open surgical repair; and absence of permanent paraplegia, disabling stroke, or dialysis that resulted from the initial operation or a secondary intervention to treat the original aortic disease.

The mid-term outcomes were late death, reintervention, conversion to OSR, and late limb or endograft occlusion.

The statistical analysis of late iliac limb occlusion rate was conducted on 132 (99.3%) patients; one patient was excluded because he underwent a complete endograft relining with an aorto-uni-iliac endograft during the EVAR procedure.

Hostile iliac artery anatomy (HIA) was defined by the presence of at least one of the following: common femoral/iliac artery diameter ≤ 7 mm, severe stenosis (≥50%) or iliac occlusion, circumferential (≥50%), and extensive (≥2 cm length) stenosis/calcification, severe angulations (≥90°), and previous surgical or endovascular repair [7]. HIA was graded as follows: Grade 1 (presence of less than two features), Grade 2 (exactly two features), or Grade 3 (more than two features).

The follow-up protocol included a duplex ultrasound examination at discharge and a contrast-enhanced CT within 30 days after surgery. The timing of the following duplex examination was at 6 months, and the following contrast CT scan was scheduled at 1 year. Thereafter, duplex scans and clinical examinations were performed annually, except in cases requiring a CT scan (e.g., persistent endoleak, short landing zone, and aneurysm growth > 5 mm).

Arterial diameters were measured on axial scans and by the same observer as the shortest outer transverse diameter of the vessel. Arterial length was measured from the CT using the centerline of flow.

Patients missing follow-up visits for more than 12 months were interviewed by telephone, while causes of death were obtained from primary care physicians, family, or death certificates.

Considering the retrospective nature of the study and the fact that all patients provided written consent for the anonymous use of their clinical data for scientific purposes, the institutional review board approval was waived for this research.

### Statistical Analysis

Data are presented as *n* (%) for qualitative variables and mean ± SD for quantitative variables. A *p*-value of < 0.05 was considered significant for all analyses. Kaplan–Meier survival estimates were calculated to assess long-term outcomes (survival, freedom from re-intervention, freedom from surgical conversion, and freedom from limb occlusion); curves are displayed up to a value of SE < 0.10. The analysis regarding iliac leg occlusions was performed separately on a population of 264 iliac legs. Statistical analysis was performed using SPSS (version 26; IBM Corporation, Armonk, NY, USA).

## 3. Results

From December 2014 to July 2023, 133 Cordis Incraft ultra-low profile endografts were implanted in 133 patients for the treatment of infrarenal abdominal aortic aneurysms, common iliac aneurysms, and abdominal penetrating aortic ulcers, both in elective and urgent settings. Patients were mostly male (91.7%) with a mean age of 75.3 ± 7.7 years. The percentages of comorbidities like hypertension, chronic obstructive pulmonary disease (COPD), heart disease, dyslipidemia, smoking, and peripheral artery disease were quite standard for a population suffering from significant atherosclerotic diseases (85.7%, 66.9%, 56.4%, 63.9%, 55.6%, and 48.1%, respectively). The population characteristics and comorbidities are reported in Table 1.

Nine patients (6.8%) were treated for symptomatic aortic aneurysm, while only one patient (0.8%) underwent an EVAR procedure with an Incraft implantation for a ruptured abdominal aortic aneurysm.

Patients’ anatomical features, obtained from the evaluation of pre-operative CT angiograms with a dedicated workstation (Aquarius Terarecon, Foster City, CA, USA), are reported in Table 2 and Table 3.

The mean axial aortic transverse diameter was 54.6 ± 8.7 mm. The proximal aortic neck’s mean diameter was 21.6 ± 3.1 mm and its mean length was 30.9 ± 1.1 mm; the mean diameter of the aortic bifurcation was 22.1 ± 5.9 mm.

In 120 cases (90.2%), at least one hostile iliac access was present, while 104 patients (78.2%) had hostility in both iliac accesses. In 48 cases (18.0%), the angulation of the iliac axis was > 90°, 133 iliac accesses (50.0%) had severe calcifications, and 127 (47.7%) had a minimum axial diameter < 7 mm. The number of iliac axes with stenosis > 50% was 125 (47.0%); 9 (3.4%) were occluded and 8 (3.0%) were aneurysmatic. The mean iliac tortuosity index was 1.44 ± 0.23 (range: 1.10–2.52). Iliac accesses with grade 1 hostility were 135 (50.8%), those with grade 2 hostility were 63 (23.7%), and those with grade 3 hostility were 68 (25.6%).

### 3.1. Intra-Procedural Outcomes

Table 4 shows the intra-operative results. Technical success was achieved in 104 patients (78.2%). There were no cases of intra-operative death or conversion to OSR. In 69 patients (51.9%), the completion angiogram showed the presence of an endoleak; 27 cases were classified as type I A, 1 case as type I B, and 41 cases as a type II endoleak (20.3%, 0.8%, and 30.8%, respectively). There were no cases of intra-operative type III, IV, or V endoleaks. The operating time was 73.0 ± 51.6 min, the fluoroscopy time was 21.2 ± 23.0 min, and 83.2 ± 37.5 mL of iodine contrast media was used.

Considering the fact that all type I endoleaks appeard as low-flow and in every case the endograft appeared positioned correctly where previosly planned, with proper oversizing, no immediate correction was considered and conservative management was chosen.

Intra-procedural complications occurred in nine patients (6.7%): six cases of surgical conversion of femoral access, one case of acute limb ischemia requiring treatment, one case of iliac rupture necessitating immediate covered stent placement, and one case of iliac leg occlusion due to incorrect positioning, treated with a femoro–femoral crossover bypass (4.5%, 3.0%, 2.3%, and 0.8%, respectively).

### 3.2. Thirty-Day Results

Table 5 illustrates the 30-day peri-operative outcomes. Clinical success within 30 days was achieved in 132 (99.3%) patients. No peri-operative or in-hospital deaths were recorded. No early surgical conversion was needed. Only one patient (0.8%) required reintervention within 30 days of the primary procedure due to an acute limb ischemia for iliac leg occlusion.

In 25 patients (18.8%), the 30-day CT angiogram showed the persistence of an endoleak, with all classified as type II endoleak. All type 1 A and 1 B endoleaks were no longer visible on the early control CT scan.

In total, 16 of the 41 intra-operative type II endoleaks were no longer visible on the 30-day control CT scan.

### 3.3. Mid-Term Results

**Figure 1:**
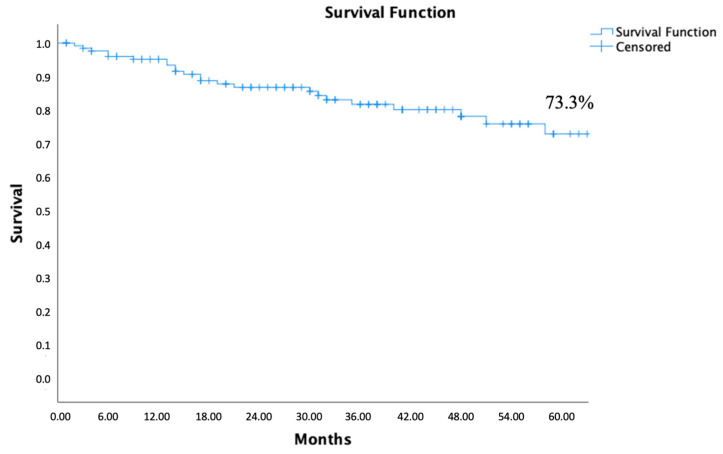
Five years overall survival estimate calculated by the Kaplan–Meier method.

The mean follow-up observational period was 35.4 ± 2.4 months (range 1–103 months). The actuarial overall survival rate estimate was 95.2% at 1 year, 82.0% at 3 years, and 73.3% at 5 years after the EVAR procedure (Figure 1).

**Figure 2:**
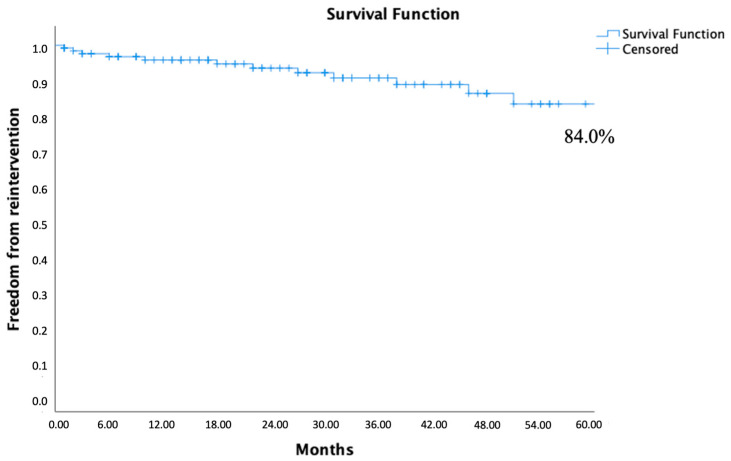
Five years of freedom from any reintervention calculated by the Kaplan–Meier method.

During the study, 13 reinterventions (9.8%) were needed in 13 patients. Reinterventions related to endograft component occlusion occurred in nine patients (6.8%): eight were treated with thrombectomy and relining for iliac leg occlusion and one was treated with an axillofemoral bypass for total endograft occlusion. Three patients (2.3%) underwent type II endoleak embolization and one patient (0.8%) required complete iliac relining due to a type III endoleak. Freedom from reintervention risk with Kaplan–Meier estimate was 96.0%, 91.1%, and 84.0% at 1, 3, and 5 years, respectively (Figure 2).

**Figure 3:**
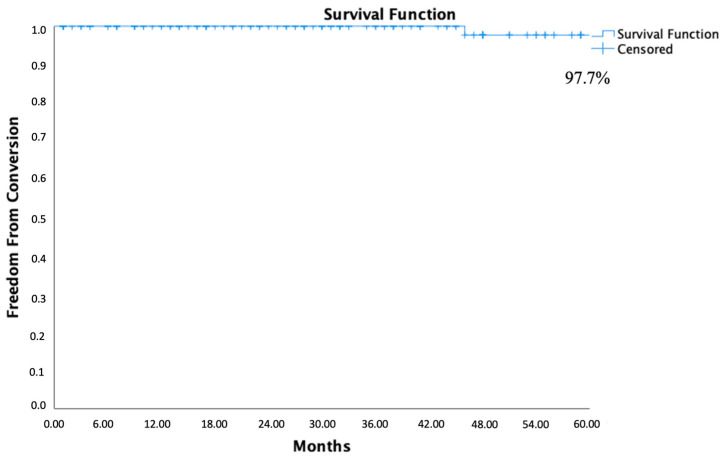
Five years of freedom from open surgical conversion calculated by the Kaplan–Meier method.

The need for late open surgical conversion occurred in only one patient (0.8%) for type I endoleak with proximal neck enlargement. Freedom from open surgical conversion risk with Kaplan–Meier estimate was 100% at 1 and 3 years and 97.7% at 5 years (Figure 3). No late endograft migration was recorded during the follow-up period.

**Figure 4:**
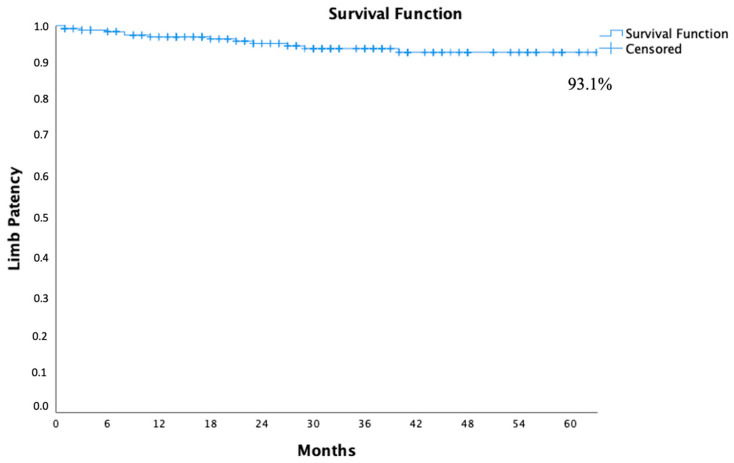
Five years of freedom from iliac leg occlusion calculated by the Kaplan–Meier method.

Overall, 14 iliac leg occlusions (5.3%) were observed: in 9 cases (6.8%), revascularization procedures were needed; the remaining 5 patients did not experience symptoms from arterial thrombosis. The actuarial patency rate was 97.1%, 94.1%, and 93.1% at 1, 3, and 5 years, respectively (Figure 4).

**Figure 5:**
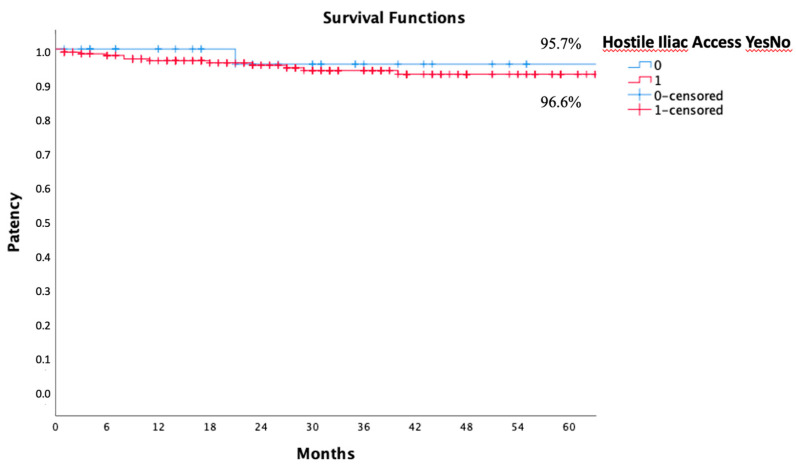
Five years of freedom from iliac leg occlusion in hostile (red) and non-hostile (blue) anatomy calculated by the Kaplan–Meier method.

A comparison between the iliac leg patency rate in hostile iliac anatomy and non-hostile anatomy showed that freedom from iliac leg occlusion risk with the Kaplan–Meier estimate was 100% at 1 year and 95.7% at 3 and 5 years in non-hostile iliac anatomies. In hostile iliac anatomies, the estimate was 99.1%, 97.2%, and 96.6% at 1, 3, and 5 years, respectively, without any statistically significant differences between the two groups (Figure 5).

**Figure 6:**
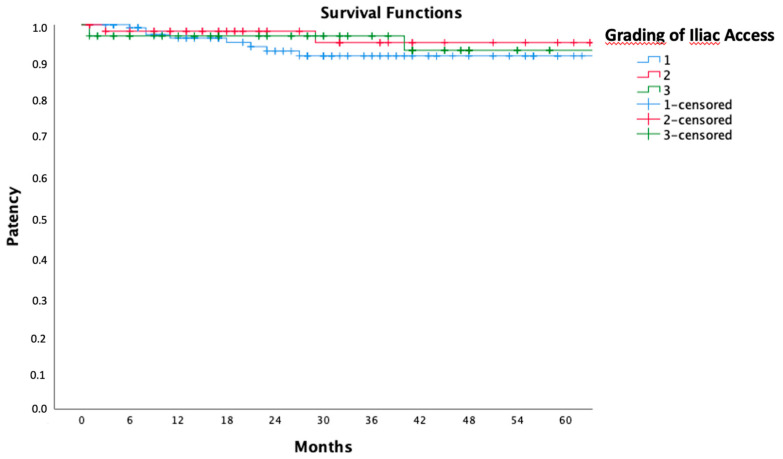
Five years of freedom from iliac leg occlusion in grade 1 (blue), grade 2 (red), and grade 3 (green) hostile anatomy were calculated by the Kaplan–Meier method.

Furthermore, an analysis of freedom from iliac leg occlusion based on iliac hostility grading was conducted (Figure 6). For HIA grade 1, freedom from iliac leg occlusion risk with the Kaplan–Meier estimate was 96.5% at 1 year and 91.9% at 3 and 5 years. For HIA grade 2, freedom from iliac leg occlusion risk was 98.3% at 1 year and 95.3% at 3 and 5 years. For HIA grade 3, freedom from iliac leg occlusion risk was 97.1% at 1 and 3 years and 93.3% at 5 years.

Statistical analysis did not demonstrate a significant difference between the three groups.

## 4. Discussion

Previous randomized comparisons and large registries [8,9,10,11,12,13,14,15] suggested that endovascular aneurysm repair (EVAR) of abdominal aortic aneurysm, compared to open surgery, might confer only a short-lasting benefit to patients since the low mortality and morbidity achieved in the perioperative period after EVAR is overcome by a late increase in AAA ruptures during follow-up. It should be noted, however, that these studies were based on the results of old-generation endoprostheses.

EVAR technology has advanced rapidly and today, many endograft models evaluated in these studies are no longer in use. Operator experience has also improved significantly, leading to clearer and more reliable indications and contraindications for EVAR, thereby allowing for better long-term results [16]. Currently, EVAR is associated with low rates of AAA-related mortality, secondary rupture, AAA growth, and need for conversion, persisting during the follow up at least in the mid-term, making it a valid alternative to open surgical repair (OSR) for an increasingly higher percentage of patients [17,18]. However, although EVAR should be considered the preferred treatment modality in most patients, it is reasonable to consider an OSR first strategy in younger fit patients with a long life expectancy of >10–15 years [2].

Despite these advancements, complex anatomical factors still represent an issue regarding the applicability and durability of the endovascular technique. Poor access remains a primary exclusion criterion for EVAR and a major cause of intra-procedural complications, with access-related complications occurring in approximately 17% of cases in some series [19]. The reduction in delivery system diameter has been a significant factor in increasing the applicability of EVAR for patients with less favorable access.

The anatomic suitability of EVAR, according to the instructions for use, of three standard bifurcated stent graft devices (Endurant^®^ [Medtronic Vascular, Santa Rosa, CA, USA], Excluder^®^ [W. L. Gore & Associates, Flagstaff, Ariz], Zenith^®^ [Cook Medical, Bloomington, IN, USA]) was evaluated by Kristmundsson et al. in 2014. They found that overall EVAR suitability was 49.4%, with the presence of narrow iliac arteries as one of the most common exclusion criteria. It has been reported that the reduction in the delivery system diameter may be able to potentially increase the overall suitability of EVAR devices by 10% [20].

**Figure 7:**
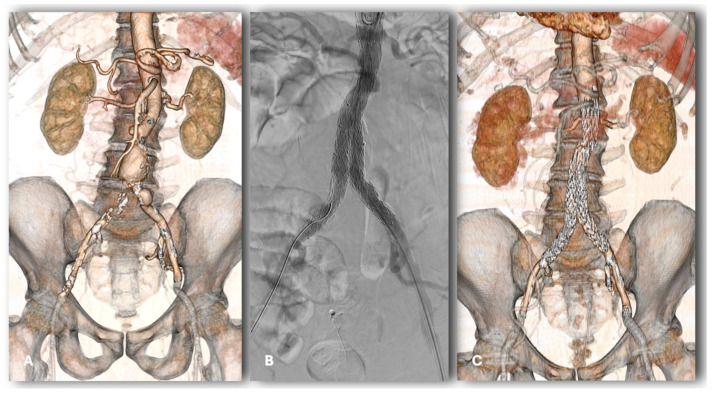
Pre-operative CT-angiogram of a patient with AAA and chronic total occlusion of the right common iliac artery (**A**); final intraoperative angiography and post-operative CT-angiogram demonstrating effective Incraft implantation with sac exclusion and complete right common iliac artery recanalization (**B**,**C**).

Ultra-low profile endografts, such as Incraft (14F outer diameter) and Ovation (Endologix, Santa Rosa, Calif), address the need to broaden the suitability of EVAR to patients with complex anatomical features, avoiding access issues and improving device deliverability (Figure 7). The safety and the effectiveness of the Incraft stent-graft have been demonstrated by the INNOVATION trial, and the 5-year data were very promising [21,22].

Data from the Triveneto Incraft Registry (TIR) showed good short- and mid-term results for the Cordis Incraft endograft, with high rates of technical success (99.5%) and freedom from reintervention during follow-up (92.1%) [23]. The most frequent endograft-related complication was iliac branch occlusion, with rates similar to other series analyzing both Incraft and other devices [24,25,26]. This is notable given that the patient population selected for treatment with this specific ultra-low-profile device had a high degree of anatomic complexity, with 86.6% having at least one severe anatomical characteristic and over 30% having two or more hostile characteristics at the iliac level.

Several studies have been published regarding the Ovation stent graft and its outcomes in patients with both standard and hostile anatomy; Greaves et al. reported a technical success rate of 100% without any case of 30-day mortality, AAA-related mortality, or iliac branch occlusion during follow-up [27].

Our study focused on a selected population of patients with hostile iliac accesses. In our center, the presence of unfavorable anatomical characteristics of the iliac axes is a criterion for the implantation of the Incraft endograft. Intraoperative technical success was achieved in 78.2% of patients, primarily due to a high percentage of low-flow type IA endoleaks seen in the final angiogram (21.1%).

In each case, the performing surgeon personally planned and conducted measurements before the procedure, carefully evaluating stent-graft oversizing. Additionally, proximal neck ballooning with a molding aortic balloon was performed before the completion of angiography. These measures, combined with the low-flow nature of proximal endoleaks, enabled conservative management with a reasonable safety margin. This approach proved effective, as type IA endoleaks were no longer detectable on the 30-day follow-up CT angiogram, resulting in a 30-day technical and clinical success rate of 99.3%, comparable to other studies on ultra-low profile endografts [23,27].

This outcome may be attributed to the structural characteristics of the Incraft endograft, which may require time to fully conform to the proximal aortic neck, or to the initial porosity of the fabric. However, these are merely hypotheses and, lacking concrete evidence, should be considered speculative. Furthermore, intraoperative complications occurred in 6.7% of patients, mostly related to vascular accesses. It can be stated, however, that these complications were somewhat predictable given the type of intervention and that the outcomes of the present study should be considered good given the anatomical complexity of the patient population.

Excluding ultra-low profile endograft availability, implanting other endoprosthesis models in patients with severe and hostile anatomical characteristics would be very complex if not impossible in some cases. According to our study, the overall survival rate estimate with Incraft endograft was 95.2% at 1 year and 73.3% at 5 years post-EVAR, with 9.8% of patients requiring reintervention during follow-up, mainly due to thrombotic occlusion of an endoprosthetic segment.

Finally, there were no statistically significant differences in freedom from iliac branch occlusion between non-hostile and hostile accesses, nor between groups with grade 1, grade 2, or grade 3 access hostility, likely due to the small number of events.

Anyway, the present study is, to the best of our knowledge, the largest single-center dataset analyzing EVAR outcomes using the Incraft endograft.

This study is limited by its single-center design. At the authors’ institution, EVAR is the first-line treatment for aortoiliac aneurysms in all cases with suitable anatomy, including young and fit patients who could potentially undergo open repair. Traditional surgery is reserved for patients with unfavorable anatomic characteristics. This approach may differ from common practices, and as a result, the outcomes presented may not be replicable in other centers. Additionally, it is a retrospective observational study, and it was not possible to compare the performance of the Incraft endograft with the performance of other endoprosthesis models implanted in patients with hostile anatomy due to selection bias, nor was it possible to randomize patients due to the retrospective nature of the study. A longer follow-up is also necessary to analyze the long-term outcomes and durability of the Incraft endograft.

## 5. Conclusions

The use of the ultra-low profile Cordis Incraft endograft represents a valid option for the endovascular treatment of AAA, especially in patients with a high grade of anatomic complexity. The procedure can be performed with satisfying rates of 30-day technical and clinical success, and the observed rates of iliac branch occlusion during follow-up can be widely justified by the anatomical features of the target population.

Further studies are required to confirm these findings and assess endograft long-term reliability.

## Figures and Tables

**Table 1:** Baseline patient characteristics.

Mean ± SD age (y) Male Smoker Hypertension Chronic obstructive pulmonary disease Ischaemic heart disease Hyperlipemia Chronic renal failure Peripheral artery disease Obesity Diabetes Previous aortic surgery Symptomatic Ruptured	75.3 ± 7.7 122 (91.7) 74 (55.6) 114 (85.7) 89 (66.9) 75 (56.4) 85 (63.9) 54 (40.6) 64 (48.1) 33 (24.8) 24 (18.0) 0 (0.0) 9 (6.8) 1 (0.8)

Note: Data are *n* (%) unless otherwise indicated.

**Table 2:** Patient anatomical features.

AAA diameter (mm) PAU, n (%) Proximal neck diameter (mm) Proximal neck length (mm) Aortic bifurcation diameter (mm) Hostility of at least 1 iliac access, n (%) Hostility of both iliac accesses, n (%)	54.6 ± 8.7 14 (10.5) 21.6 ± 3.1 30.9 ± 1.1 22.1 ± 5.9 120 (90.2) 104 (78.2)

Note: Data are mean ± SD unless otherwise indicated. AAA = abdominal aortic aneurysm.

**Table 3:** Iliac anatomical features.

Iliac angulation > 90° Severe calcification External iliac diameter < 7 mm Iliac stenosis Iliac occlusion Common iliac aneurysm Iliac tortuosity index, mean ± SD Grade 1 hostility Grade 2 hostility Grade 3 hostility	48 (18.0) 133 (50.0) 127 (47.7) 125 (47.0) 9 (3.4) 8 (3.0) 1.44 ± 0.23 135 (50.8) 63 (23.7) 68 (25.6)

Note: Data are *n* (%) unless otherwise indicated. N = 266.

**Table 4:** Intraoperative results.

Technical success Intraoperative death Intraoperative conversion to OSR Intraoperative endoleak Type 1A endoleak Type 1B endoleak Type II endoleak Type III endoleak Type IV endoleak Type V endoleak Other complications Access surgical conversion Lower limb ischemia Iliac rupture Iliac branch occlusion	104 (78.2) 0 (0.0) 0 (0.0) 69 (51.9) 27 (20.3) 1 (0.8) 41 (30.8) 0 (0.0) 0 (0.0) 0 (0.0) 9 (6.7) 6 (4.5) 1 (0.8) 1 (0.8) 1 (0.8)

Note: Data are *n* (%) unless otherwise indicated. OSR = open surgical repair.

**Table 5:** Thirty-day results.

Clinical success Death Reintervention Conversion to open surgical repair Endoleak Type I endoleak Type II endoleak Type III endoleak Type IV endoleak Type V endoleak	132 (99.3) 0 (0.0) 1 (0.8) 0 (0.0) 25 (18.8) 0 (0.0) 25 (18.8) 0 (0.0) 0 (0.0) 0 (0.0)

Note: Data are *n* (%) unless otherwise indicated.

## Data Availability

Data are contained within the article.
